# A Review of Outcomes and Technique for the Robotic-Assisted Laparoscopic Retroperitoneal Lymph Node Dissection for Testicular Cancer

**DOI:** 10.1155/2018/2146080

**Published:** 2018-05-03

**Authors:** Zeyad R. Schwen, Mohit Gupta, Phillip M. Pierorazio

**Affiliations:** Department of Urology, James Buchanan Brady Urological Institute, Johns Hopkins University School of Medicine, Baltimore, MD, USA

## Abstract

**Objectives:**

The robotic-assisted laparoscopic retroperitoneal lymph node dissection (R-RPLND) represents a new frontier in the surgical management of testicular cancer in the realm of minimally invasive urologic oncology. We aimed to review the early outcomes as compared to the laparoscopic and open approaches as well as describe the operative technique for the R-RPLND.

**Materials and Methods:**

We reviewed all the literature related to the R-RPLND based on an electronic PubMed search up until July 2017.

**Results and Discussion:**

Encouraged by favorable early oncologic and safety outcomes for treatment of clinical stage (CS) I nonseminomatous germ cell tumor (NSGCT), the R-RPLND affords the same recovery advantages as the laparoscopic retroperitoneal lymph node dissection (L-RPLND) while offering greater dexterity, superior visualization, and a theoretically shorter learning curve for the surgeon. While R-RPLND has a promising future in the management of patients with primary and postchemotherapy NSGCT, larger and more vigorous prospective studies are needed before supplanting the open RPLND as the gold standard approach for primary low-stage NSGCT or becoming an equivalent surgical modality in the postchemotherapy setting.

## 1. Introduction

Testicular germ cell tumor (GCT) is the most common solid tumor in men between the ages of 20–44. Men diagnosed with GCT have excellent survival rates due to advances in the multimodal treatment paradigm of chemotherapy, radiation therapy, and surgery [1]. Retroperitoneal lymph node dissection (RPLND) remains an established treatment option for nonseminomatous GCT in the primary setting for low-stage (clinical stages (CSs) I and II) diseases and residual masses after chemotherapy [1]. Due to the excellent survival outcome, with a 5-year overall survival rate of 98%, there has been a greater emphasis on reducing morbidity and long-term toxicity for testicular cancer survivors. Open RPLND (O-RPLND) remains the gold standard approach for surgical management of the retroperitoneum for GCTs. It is, however, maximally invasive and can result in significant postoperative morbidity and prolonged hospitalizations [2–4].

For primary CS I and CS II NSGCT, minimally invasive RPLND has become a less morbid alternative to the O-RPLND while touting favorable early oncologic outcomes [5, 6]. Laparoscopic RPLND (L-RPLND), first reported in 1992, offered a reduced recovery time, less blood loss, and lower complication rates compared to O-RPLND [5, 7]. However, the operation had a very steep learning curve [8], a lower lymph node yield [9], and critics have pointed out that the L-RPLND's long-term oncologic outcomes have not been studied as rigorously as O-RPLND, which is partly related to the high rate of adjuvant chemotherapy given to patients with positive lymph nodes [10].

The natural evolution of minimally invasive urologic oncology from laparoscopy to robotics laid the foundation for the first robotic-assisted laparoscopic RPLND (R-RPLND) to be performed in 2006 by Davol et al. [11]. The advantages of R-RPLND over L-RPLND were similar to other urologic operations that transitioned to a robotic approach: a reduced learning curve, three-dimensional visualization, and greater instrument dexterity from the wristed instruments. Boosted by early results demonstrating equivalence in oncologic and safety measures compared to open and laparoscopic approaches, the R-RPLND has become an excellent option for the treatment of CS I and CS II nonseminomatous GST (NSGCT) and is emerging as a feasible approach for postchemotherapy RPLND [12]. In this article, we will review the technique and early outcomes of R-RPLND as compared to both O-RPLND and L-RPLND for the management of primary low-stage nonseminomatous and postchemotherapy GCT.

## 2. Materials and Methods

We performed an electronic PubMed search for all relevant publications regarding the outcomes and technique of the R-RPLND up until July 2017. We used the keywords robotic, retroperitoneal lymph node dissection, and testicular cancer, which resulted a total of 36 papers. All single and multi-institutional R-RPLND studies in adults with testicular cancer were included and reviewed in addition to studies investigating outcomes associated with O-RPLND and L-RPLND.

## 3. Results and Discussion

### 3.1. Primary Low-Stage Nonseminomatous Testicular Cancer

#### 3.1.1. Role of RPLND in the Guideline-Directed Management of Low-Stage NSGCT

Patients diagnosed with CS I NSGCT, based on NCCN guidelines, have the option of active surveillance, platinum-based chemotherapy, or RPLND [1]. While each treatment option offers an excellent survival rate, each has its own respective drawbacks as well. Active surveillance offers the best opportunity to avoid unnecessary treatment and is the preferred treatment based on NCCN guidelines for CS IA disease; however, patients with recurrence are often subject to three or four cycles of platinum-based chemotherapy, and not all patients are willing to accept the anxieties associated with surveillance [13]. Adjuvant chemotherapy offers the best cure rate as a single modality, approaching 97%; however, it will overtreat a significant number of men and subject them to the known and unknown long-term toxicities of platinum-based chemotherapy [14]. These long-term toxicities include secondary malignancy, early cardiovascular disease, and a number of single-organ toxicities, including nephrotoxicity, pulmonary toxicity, ototoxicity, neurotoxicity, and hypogonadism [15]. Primary RPLND offers the ability to accurately stage the extent of disease while avoiding the significant toxicity of chemotherapy [16, 17] or the high relapse rate that is observed in 20–30% of patients who choose surveillance [13, 18]. While RPLND for CS I NSGCT may result in overtreatment for some patients, 25–35% of patients will harbor metastatic disease on presentation without radiographic evidence of pathologic retroperitoneal lymph nodes [19]. When RPLND is performed at a high-volume institution, the risk of recurrence is low (2%) with survival rates exceeding 95% [20]. From the open experience, RPLND alone is curative in 80–90% of patients with pN1 disease discovered in the retroperitoneum [21]. The disadvantages of RPLND are the risk of complications, which include ejaculatory dysfunction, blood loss, visceral injuries, ileus, and chylous ascites [4]. In the phase III randomized study of primary chemotherapy versus RPLND, the recurrence rate was higher in the RPLND group (8% versus 0.5%), but 37% of patients undergoing chemotherapy experienced a grade III or IV toxicity, compared to only 9% of RPLND patients [22].

## 4. Outcomes

### 4.1. Oncologic Outcomes

While the first use of the L-RPLND was for the purpose of staging alone, the intent of the R-RPLND is to match the oncologic efficacy of O-RPLND while providing the benefits of a minimally invasive approach. The largest R-RPLND series to date, a multi-institutional study of 47 CS I and 5 CS II patients which includes our patient experience, reported an excellent 2-year recurrence-free survival of 97% in the entire cohort [6]. Stepanian et al., with a median follow-up of 49 months, reported no retroperitoneal or distant recurrences in 19 patients undergoing robotic RPLND [23]. These results are summarized in Table 1. Impressively, 75% (6/8) of patients with positive retroperitoneal lymph nodes received no additional therapy while two patients with elements of embryonal carcinoma in their retroperitoneum on final pathology received chemotherapy. It is worth noting that three of these patients had teratoma who were definitively treated with surgery alone and were not candidates for chemotherapy. Pearce et al. reported that 62% (5/8) of patients with positive lymph nodes received chemotherapy with a single out-of-template recurrence of teratoma after chemotherapy [6]. These oncologic results compare favorably to O-RPLND and L-RPLND which, based on the findings from a large meta-analysis of >800 patients, experience recurrence-free rates of 92.5% and 95.4%, respectively [9]. While the results are promising, the low rates of positive lymph nodes in these series, ranging from 17% to 42%, and the use of adjuvant chemotherapy for node-positive patients make drawing conclusions, regarding comparative efficacy to O-RPLND and L-RPLND, a challenge. Furthermore, the majority of these studies investigating R-RPLND have short follow-up and varying surgical techniques, which further complicates their collective analysis.

Lymph node yield, a correlate for the extent of node dissection, can provide valuable information regarding the staging and therapeutic benefit of R-RPLND. Importantly, their reported median lymph node yield (LNY) of 26 nodes (IQR I8-32) outperformed the LNY reported in a contemporary meta-analysis of L-RPLND, which reported a median of 16 lymph nodes [9]. R-RPLND, however, appears to be similar to LNY observed in O-RPLND, which ranges from 28–33 [24, 25]. Conversely, in a separate head-to-head comparison from our institution of 16 R-RPLNDs and 21 L-RPLNDs from a single-surgeon experience, no difference in the LNY was found [26]. To date, no prospective R-RPLND series has been published, and long-term oncologic and survival outcomes have yet to be reported in a large series. While the early results are promising and appear to suggest favorable recurrence rates and LNY compared to L-RPLND and O-RPLND in the hands of experienced robotic surgeons, determining oncologic equivalency to O-RPLND and L-RPLND will require larger, prospective series with longer follow-up.

Furthermore, though the oncologic outcomes of the primary RPLND for the management of low-stage NSGCT are often deliberated, there is considerable agreement that RPLNDs should be performed exclusively by experienced high-volume surgeons at experienced institutions, which result in fewer complications and superior oncologic outcomes [20, 27]. The early published oncologic outcomes of the R-RPLND, it is worth noting, are from experienced robotic surgeons in high-volume academic centers.

### 4.2. Perioperative Outcomes

As in other minimally invasive surgeries in urologic oncology, the R-RPLND affords a reduced blood loss and shorter recovery time, both of which translate into shorter hospital stays [28]. Blood loss is minimized for R-RPLND primarily due to the tamponading effects of pneumoperitoneum on venous bleeding. For R-RPLND, Harris et al. demonstrated equivalent blood loss (75 mL, IQR 50–100 mL) and operative time (270.5 minutes (mins), IQR 236–299 mins) compared to L-RPLND [26]. Similarly, two other series reported a median blood loss of 50 mL [6, 23], significantly less than the reported 184–450 mL blood loss for open primary RPLND [4, 29–31].

Perhaps, the most significant advantage that is afforded by the R-RPLND is the shorter recovery time compared to O-RPLND that translates into a shorter hospital length of stay (LOS). Pearce et al. and Stepanian et al. both reported a median LOS of 1 day which was far superior to both L-RPLND and O-RPLND (3.3 days and 6.6 days, resp.) [6, 9, 23]. Cheney et al., in a smaller series of 10 patients with low-stage NSGCT who received a R-RPLND, experienced a similarly short 2.7 day LOS [32]. Some O-RPLND series at experienced high-volume centers, however, have managed to reduce the difference in LOS compared to minimally invasive approaches. Syan-Bhanvadia et al. via the extraperitoneal open approach and Beck et al. of Indiana University have reported a mean LOS of 2.8 to 3 days [30, 31]. The dramatically reduced hospitalization of R-RPLND is likely explained by both the less morbid incision compared to O-RPLND and the lower rates of postoperative ileus. Together, this translates into a shorter convalescence due to less pain, earlier ambulation, and earlier return of bowel function.

Minimally invasive approaches for RPLND, however, are limited by a greater operative time compared to O-RPLND, which persists even beyond the learning curve [9]. In a large meta-analysis, L-RPLND performed by experienced surgeons had significantly greater operative time compared with O-RPLND (204 mins versus 186 mins) [9]. A similar trend applies to R-RPLND, reporting greater operative times ranging from 239 to 311 minutes [6, 26, 32]. While there are no studies investigating costs associated with R-RPLND, prior studies have demonstrated that the reduced LOS associated with L-RPLND drove its reduced cost relative to O-RPLND [33]. While the cost of robotic technology may be high relative to laparoscopy, cost savings for R-RPLND may be achieved through shorter length of stays and reduced complication rates, as was shown for laparoscopic versus robotic partial nephrectomy [34].

### 4.3. Complications

As part of the rationale for primary RPLND to avoid the long-term toxicities of chemotherapy, surgical complications need to be minimized at all costs. O-RPLND, however, has traditionally experienced relatively high intraoperative and postoperative complication rates at 5–7% and 24–33%, respectively [2, 4]. More contemporary O-RPLND series, however, report lower overall complication rates as low as 7% for primary RPLND [35]. The majority of serious intraoperative complications represent visceral injury or bleeding from lumbar veins or the great vessels which may require transfusions or, rarely, open conversion. Pearce et al. reported only two (4.3%) intraoperative complications, one of which was due to an aortic injury requiring open conversion for vascular repair. Conversion rates reported in the literature for L-RPLND are similarly rare (3.7%, range 1–5.4%) [9].

Postoperative complication rates are similarly low for R-RPLND compared to open and laparoscopic approaches. Pearce et al. reported only two Clavien Grade 1 complications and two Clavien Grade 3 complications for a postoperative complication rate of 8.5% [6]. While making direct comparisons is challenging, it appears that postoperative complications for R-RPLND are congruent to large series of L-RPLND and O-RPLND, reporting complication rates of 15.5% [9] and 7–33% [4, 35], respectively. Of note, R-RPLND experienced dramatically fewer instances of postoperative ileus compared to open series (2% versus 18%) [4, 6]. This is likely related to differences in the technique of bowel mobilization. Interestingly, two of four complications reported by Pearce et al. were chylous ascites (4.3%), which is significantly greater than the 0.4%–1.7% rate reported in a primary open series [4, 9, 36]. This complication includes only two patients and may represent a statistical anomaly due to the small cohort size or the early learning curve; however, it cannot be ignored and it warrants further consideration in future R-RPLND series. The rate of chylous ascites for R-RPLND, however, appears to be an improvement over L-RPLND, which has published rates as high as 6.6% [9, 37]. Proponents of R-RPLND believe that the improved dexterity and visualization facilitates superior ligation of lymphatics relative to L-RPLND.

The vast majority of low-stage NSGCT patients should obtain a nerve-sparing procedure to preserve antegrade ejaculation for reducing the morbidity of long-term sexual dysfunction. Pearce et al. reported 100% preservation of antegrade ejaculation [6]. Similar excellent functional outcomes are reported in the smaller R-RPLND series. In a comparative series to L-RPLND, 11% of patients who underwent a laparoscopic procedure experienced ejaculatory dysfunction compared to 0% in the robotic cohort [26]. Cheney et al. also reported a preservation of antegrade ejaculation in 10 of 11 patients [32].

## 5. Primary RPLND Technique

### 5.1. Intraoperative Technique

R-RPLND for primary CS I NSGCT involves a transperitoneal approach with the patient typically positioned in the modified flank position with a slightly flexed table. After pneumoperitoneum is achieved with a Veress needle, a 12 mm camera port and three 8 mm robotic ports are placed in a standard linear fashion to triangulate the retroperitoneum. Typically, a 12 mm AirSeal and a 5 mm blunt assistant port are also placed. Others have described a supine or dorsal lithotomy positioning with the patient placed in Trendelenburg and robotic docking occurring over the patient's left shoulder for the daVinci Si or alongside the patient for the daVinci Xi using a four-port linear configuration [23]. A major advantage to supine positioning is a more convenient shift to a bilateral template without repositioning the patient upon either identifying positive LNs on frozen sectioning for primary RPLNDs or in the postchemotherapy setting. A modified node template dissection, including nerve sparing, is performed as previously described [38–40]. A unilateral template may be performed for CS I disease, and a bilateral template is recommended for CS II disease [41]. After reflection of the ipsilateral colon to reveal the retroperitoneum, dissection is performed following boundaries of the renal vein superiorly, the ureter laterally, and the iliac bifurcation inferiorly. The gonadal vein is identified and ligated at the level of its origin, and the remaining portion of the ipsilateral spermatic cord is dissected free from the inguinal ring. For a left-sided template, lymph node packets are removed from the left common iliac nodes, preaortic, paraaortic, and retroaortic areas. Right sided-templates include lymph nodes from the paracaval, interaortocaval, and preaortic spaces. The sympathetic chain and postganglionic nerve fibers are identified and preserved. Hem-o-lok clips are placed on lymph node packets for preventing postoperative lymphatic leak as well as for control of lumbar vessels. Retrocaval and retroaortic lymph node packets can be more challenging to manage and require special consideration to ensure a complete lymph node dissection. Lumbar vessels are ligated using a variety of techniques, including surgical clips, ties, or suture ligation. The dexterity of robotic instruments allows for complete control of the great vessel to ensure dissection of all retrocaval or retroaortic tissue. In addition, the magnified view facilitates nerve dissection and preservation; the camera angle often allows a unique view of retrocaval and retroaortic structures. After hemostasis is achieved, a fibrin sealant may be applied to the lymphatic beds to prevent lymphatic leaks. A drain is typically not placed.

### 5.2. Postoperative Care

Patients are transferred to the floor where their diet is advanced from clear liquids on the night of surgery to a fat-free regular diet on the day after surgery. It is our practice to follow a low-fat diet that is regularly advanced over four weeks to minimize the risk of chyle leak. There is a paucity of data regarding the efficacy of this approach; however, we have not experienced a chyle leak in our institutional experience with minimally invasive RPLND following this protocol. Patients receive education from a nutritionist regarding a low-fat diet (≤20 g fat/day), which they will continue for 4 weeks postoperatively. Patients are usually discharged home on postoperative day 1 after they are tolerating a regular diet and have successfully ambulated and voided with adequate pain control. Patients usually return to school or work within 2 weeks.

## 6. Postchemotherapy RPLND

Postchemotherapy RPLND (PC-RPLND) for patients with residual tumors after chemotherapy represents a far more challenging surgery compared to the primary RPLND setting. Desmoplasia from the therapeutic action of chemotherapy can fuse normal tissue planes and add complexity to dissection of tissues and, if needed, repair of vascular injuries. The risk-benefit ratio of cancer cure and morbidity of surgery makes justification of an investigational, minimally invasive technique more challenging. However, select surgeons and centers report perioperative outcomes supporting minimally invasive PC-RPLND as a safe surgery. In addition, perioperative outcomes and complications, including the preservation of antegrade ejaculation, are significantly worse due to the need for a bilateral template and the treatment effect of chemotherapy on the retroperitoneal tissue [3, 4]. Based on the favorable outcomes of L-RPLND in the postchemotherapy setting [42], the natural evolution of the R-RPLND has included attempts by experienced robotic surgeons to perform postchemotherapy R-RPLND (PC-RPLND) in selected patients. To date, only two smaller series have demonstrated early feasibility [12, 32]. The Mayo Clinic R-RPLND experience included nine patients who were postchemotherapy. Notably, their median LNY (18 nodes), blood loss (313 mL), and LOS (2.2 days) were not significantly different from their primary R-RPLND patients; however, their PC-RPLND patients experienced significantly greater operative time (369 mins versus 311 mins, *p*=0.03). Notably, two patients required open conversion in the postchemotherapy group, which represents a conversion rate of 22.3%. At a median follow-up of 22 months, there were no retroperitoneal recurrences. Kamel et al., in a series of 12 patients, experienced a 91.7% completion rate with only a single open conversion due to what the author considered poor patient selection [12]. It is also worth mentioning that Stepanian et al. included four robotic PC-RPLNDs with no conversions [23]. At a median follow-up of 31 months, there were no recurrences. Currently, the literature on robotic PC-RPLND outcomes is immature and requires larger series before conclusions regarding oncologic and safety performance can be made. From these smaller series, we can conclude that R-RPLND in the postchemotherapy setting is feasible with an understandably higher rate of open conversion. Postchemotherapy RPLND has inherent objectives that are different from the primary RPLND setting, and incomplete control of the retroperitoneum or an incomplete resection during RPLND is a predictor of worse survival in the postchemotherapy setting [43]. Therefore, it is our position that oncologic outcomes remain the priority in the postchemotherapy setting, and oncologic outcomes should not be leveraged against perioperative outcomes. As larger, multi-institutional cohorts are published, we can hopefully better evaluate the merits and technique of a robotic PC-RPLND.

## 7. Conclusion

The first L-RPLND performed in 1992 and the first R-RPLND performed in 2006 marked the beginnings of a minimally invasive era to reduce the treatment morbidity for testicular cancer survivors. Early results from expert robotic surgeons at high-volume academic institutions have demonstrated both feasibility as well as favorable early oncologic outcomes and complication rates in the primary RPLND setting compared to O-RPLND and L-RPLND. Larger, prospective studies are required to better evaluate long-term oncologic outcomes and complication rates in both the primary and postchemotherapy settings.

## Figures and Tables

**Table 1:** Summary of perioperative outcomes from notable series for primary and postchemotherapy R-RPLND.

Primary RPLND													
Group	Year	*N*	CS I	CS II	Operative time (mins)	EBL (ml)	Lymph node yield	LOS (days)	Complication rate (%)	Positive lymph nodes (%)	Recurrence-free rate (%)	Antegrade ejaculation (%)	Follow-up (months)

Harris et al. [26]	2015	16	16	0	294	75	22	—	6.3	12.5	—	100	13.5
Cheney et al. [32]	2015	10	9	1	311	100	22	2.75	—	30	80	91	22
Stepanian et al. [23]	2016	16	11	5	293	50	19.5	1	5	38	100	90	49
Pearce et al. [6]	2017	47	42	5	235	50	26	1	14	17	97	96	16

Postchemo RPLND												
Group	Year	*N*	CS I	CS II	Operative time (mins)	EBL (ml)	Lymph node yield	LOS (days)	Complication rate (%)	Positive lymph nodes (%)	Recurrence-free rate (%)	Antegrade ejaculation (%)	Follow-up (months)

Cheney et al. [32]	2015	8	7	1	369	313	18	2.2	—	62.5	100	—	22
Stepanian et al. [23]	2016	4	1	3	324	150	22	1.5	—	50	100	100	41
Kamel et al. [12]	2016	12	6	6	312	475	22	3	25	50	100	66.7	31

LOS = length of stay; EBL = estimated blood loss; CS = clinical stage.
